# Acute Traumatic Aortic Injury: What the Radiologist Needs to Know

**DOI:** 10.3390/tomography12040057

**Published:** 2026-04-13

**Authors:** Kristina Ramirez-Garcia, Catalina Jaramillo, Emma Ferguson, Jason Au, Erika Odisio, Gustavo S. Oderich, Daniel Ocazionez, Cihan Duran, Thanila Macedo

**Affiliations:** 1 Department of Diagnostic and Interventional Imaging, McGovern Medical School, The University of Texas Health Science Center at Houston, Houston, TX 77030, USA; emma.c.ferguson@uth.tmc.edu (E.F.); jason.m.au@uth.tmc.edu (J.A.); erika.g.odisio@uth.tmc.edu (E.O.); daniel.ocazioneztrujillo@uth.tmc.edu (D.O.); cihan.duran@uth.tmc.edu (C.D.); 2 Department of Radiology and Diagnostic Imaging, Ochsner Health, New Orleans, LA 70121, USA; catalina.jaramillofranco@ochsner.org; 3 Division of Vascular Surgery and Endovascular Therapy, Michael E. DeBakey Department of Surgery, Baylor College of Medicine, Houston, TX 77030, USA; gustavo.oderich@bcm.edu; 4 Department of Radiology, Baylor College of Medicine, Houston, TX 77030, USA; thanila.macedo@bcm.edu

**Keywords:** traumatic aortic injury, imaging, computed tomography angiography

## Abstract

Acute traumatic aortic injury (ATAI) is a rare but life-threatening consequence of blunt trauma that requires prompt diagnosis and accurate imaging assessment. This review presents an imaging-based approach to ATAI, with emphasis on computed tomography angiography (CTA) as the first-line modality for diagnosis, grading, treatment planning, and follow-up. CTA enables the detection of both direct and indirect signs while also allowing for the assessment of lesion severity, extent, and associated findings that may influence management. Familiarity with common mimics and anatomic variants improves diagnostic confidence and helps avoid false positive interpretations. Careful protocol optimization, including multiphasic acquisition, bolus timing, and postprocessing reconstructions, can further enhance image quality and diagnostic performance. Recognition of patient-related and technical CTA artifacts, along with strategies to reduce them, including the selective use of ECG-gated CTA, may further decrease diagnostic uncertainty. We also discuss the complementary roles of emerging CT technologies and magnetic resonance angiography in selected patients. Finally, we review current classification systems, imaging-guided management, post-treatment surveillance, and potential complications. Awareness of ATAI imaging findings, protocol optimization, and diagnostic pitfalls is essential for accurate interpretation and effective multidisciplinary care.

## Simple Summary

Acute traumatic aortic injury is an uncommon but often fatal consequence of blunt trauma. This review explains how computed tomography angiography helps doctors quickly detect injury, judge its severity, avoid common false alarms, guide treatment, and monitor patients after repair. It also highlights why scan techniques, image reconstruction, and awareness of normal variants and artifacts matter in everyday practice. By bringing together these imaging features and practical considerations, this review may support faster, more accurate care and help guide future studies on imaging protocols, risk assessment, and long-term follow-up.

## 1. Introduction

Acute traumatic aortic injury (ATAI) is a rare but highly lethal consequence of blunt trauma and remains the second leading cause of death following blunt injury [1]. Prehospital mortality is extremely high, approaching 80–90%. Although survival improves among patients who reach the hospital, outcomes remain strongly influenced by associated critical injuries [2,3,4]. Given this high mortality, rapid diagnosis and triage are essential, placing imaging at the center of ATAI evaluation and management.

Contemporary contrast-enhanced computed tomography angiography (CTA) has transformed the assessment of ATAI. It enables rapid and accurate detection of direct and indirect signs of aortic injury, precise localization and grading, and evaluation of associated injuries. Imaging findings directly inform clinical decision making, the urgency of intervention, and the choice between conservative management and endovascular repair.

This review focuses on the imaging-based diagnosis, evaluation, and follow-up of ATAI, with emphasis on CTA and complementary imaging modalities. Relevant background on pathophysiology and classification is also included to support interpretation of the imaging findings and related management decisions.

## 2. Pathophysiology

ATAI results from a combination of mechanical forces generated by rapid deceleration, pressure changes, and osseous compression that disrupt the aortic wall [5]. Many of these biomechanical factors are present in motor vehicle collisions.

**Figure 1:**
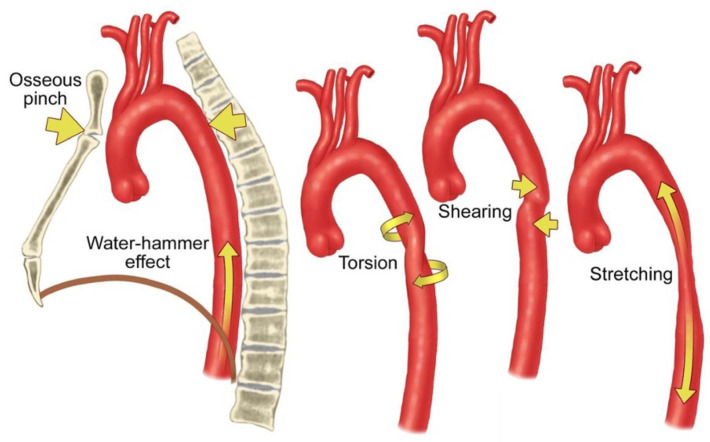
Biomechanical factors involved in aortic injury. The illustration depicts the mechanical forces contributing to aortic injury following rapid deceleration. Arrows indicate the direction of the applied forces and vessel deformation associated with osseous pinch, water-hammer effect, torsion, shearing, and stretching Source: This figure was created by the authors.

Sudden deceleration causes aortic segments to shift abruptly relative to fixed anatomic structures. The aortic isthmus, located between the left subclavian artery and the ligamentum arteriosum, is particularly susceptible to injury because it is a relatively immobile section of the aorta. It is the most frequently injured region in ATAI [4,5,6]. The sinotubular junction and the origin of the left subclavian artery are also vulnerable to pressure changes resulting from thoracic acceleration and peak wall stress. Rapid deceleration generates complex mechanical forces, including the water hammer effect (hydraulic shock), torsion, shear, and stretch that can cause aortic rupture or dissection (Figure 1) [7]. The osseous pinch mechanism occurs when the aorta is compressed between the spine and anterior bony structures. Additional factors, such as aortic arch morphology, blood pressure, and the direction of force, can increase the risk of ATAI [8].

## 3. Imaging Diagnosis

Multiple imaging modalities may be used to assess patients after trauma, with the initial imaging approach guided by clinical suspicion and hemodynamic stability. A supine chest radiograph is often the first imaging study obtained in the emergency department, as it may help identify other life-threatening conditions. However, it is not a reliable screening tool for suspected aortic injury [9]. Its sensitivity for identifying ATAI is highly variable (81–100%), and its specificity remains limited (34–60%) [9,10,11,12,13]. The review of the chest radiograph should not delay cross-sectional imaging when ATAI is suspected [14,15]. Computed tomography (CT) is used for definitive diagnosis, classification, and management planning because of its superior diagnostic accuracy. Conventional angiography now generally provides no additional benefit for occult intimal injury. Additional imaging modalities are rarely used for diagnosis but may be helpful in select cases.

### 3.1. Chest CT Angiography

Chest CTA is the first-line imaging modality for the evaluation of suspected ATAI. It is widely available, cost effective, rapidly acquired, and offers high spatial resolution with good patient tolerance. The reported sensitivity and specificity for detecting traumatic aortic injury approach 100% when a modern multidetector CTA is used [10,16]. It provides a lower threshold for detecting active hemorrhages than digital subtraction angiography (0.35 vs. 0.5 mL/min) [17]. Beyond diagnosis, CTA plays a central role in injury grading, risk stratification, management planning, and post-intervention assessment, making it indispensable throughout the continuum of ATAI care [18].

#### 3.1.1. Direct Signs of Aortic Injury

**Figure 2:**
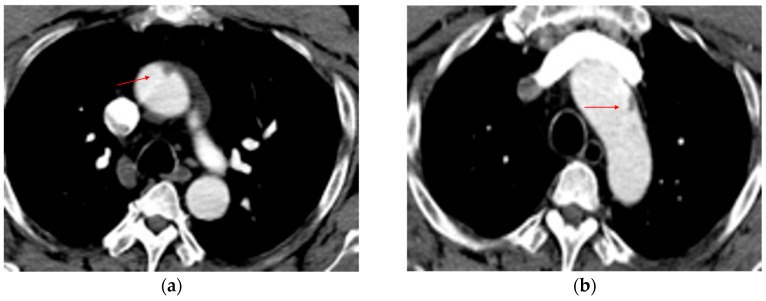
Intimal flap. (**a**,**b**) Axial CTA images show a small filling defect adherent to the aortic wall (red arrows).

**Figure 3:**
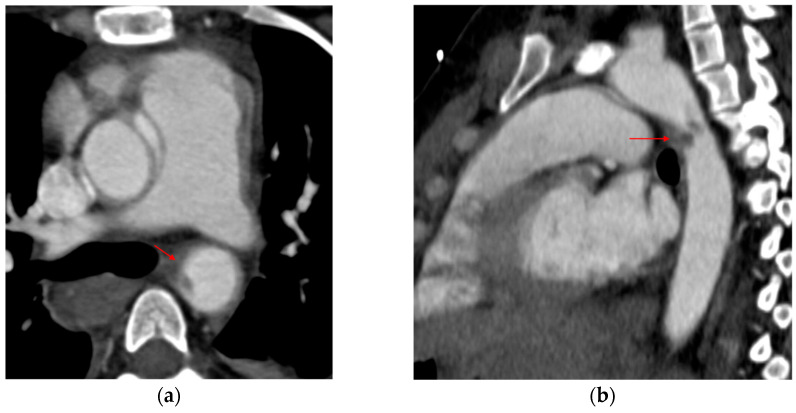
Intramural hematoma. (**a**) Axial and (**b**) sagittal CTA images demonstrate focal wall thickening (red arrows) along the anterolateral aortic wall, with an associated small intraluminal filling defect.

**Figure 4:**
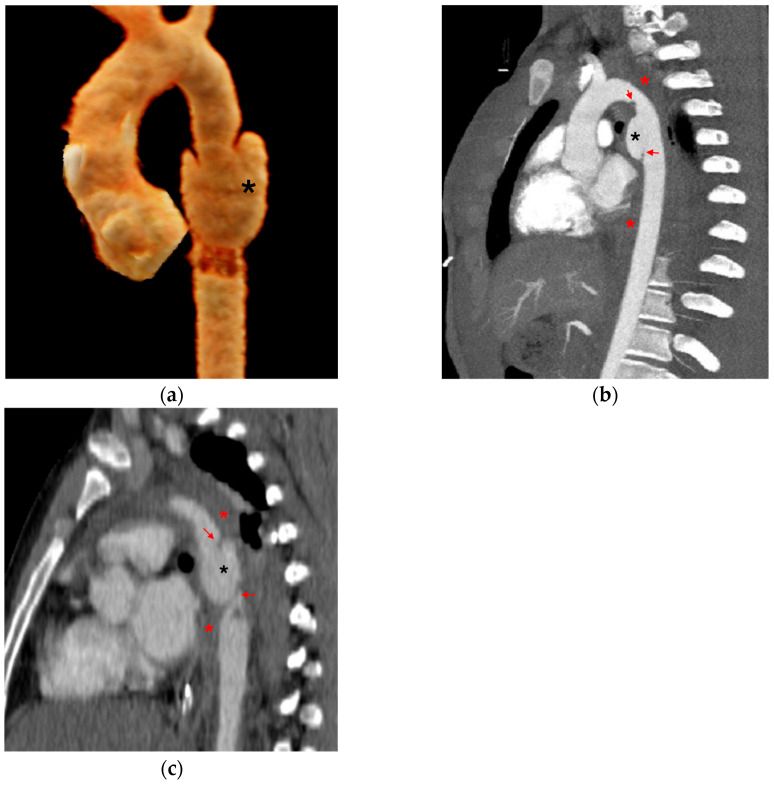
Pseudoaneurysm. (**a**) 3D volume-rendered and (**b**,**c**) sagittal CTA images show focal dilatation (black asterisks) and intimal disruption (red arrows) along with a periaortic hematoma (red asterisks).

**Figure 5:**
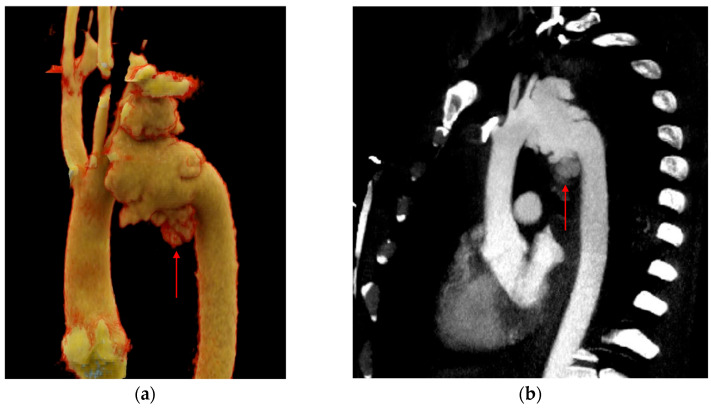
Contrast extravasation. (**a**) 3D volume-rendered and (**b**) sagittal CTA images of the chest demonstrate aneurysmal dilatation at the distal arch with extravasation of contrast beyond the aortic contour (red arrows).

Direct signs include changes in the normal appearance of the aorta, as well as injury to the intima, media, or adventitia, each of which has characteristic radiologic features [15,19,20]. An *intimal flap* occurs when the inner layer of the aorta tears. On CT, it appears as a linear filling defect in the aortic lumen (Figure 2). Intimal flaps visible in the axial plane may only be seen on one or two slices and can be confirmed on coronal or sagittal views. An *intraluminal thrombus* appears as a rounded filling defect within the aorta, indicating clot formation after intimal injury. An *intramural hematoma (IMH)* appears as a crescent-shaped density in the aortic wall, indicating blood accumulation (Figure 3). IMH results from rupture of the vasa vasorum in the medial layer of the aortic wall or from an intimal tear that allows blood to enter the wall and thrombose because of stasis [21]. A *pseudoaneurysm* is a bulge that produces an abnormal aortic wall contour (Figure 4). It contains blood but is not lined by all three layers of the aorta; the intima and media are disrupted, whereas the adventitia remains intact. If left untreated, the adventitia may rupture. A pseudoaneurysm/normal aortic diameter ratio > 1.4 is a predictor of rupture [22]. *Contrast extravasation* occurs when contrast material leaks from the aorta into the surrounding tissues, signaling active bleeding (Figure 5). *Periaortic contrast extravasation* is an indicator of a full-thickness tear in the aorta, but its absence does not rule out transection, as contrast may remain contained within the periaortic soft tissue even in cases of complete transection. These cases are uncommon, as patients are often too unstable or do not survive to reach the hospital [10,15,19,20].

#### 3.1.2. Indirect Signs of Aortic Injury

**Figure 6:**
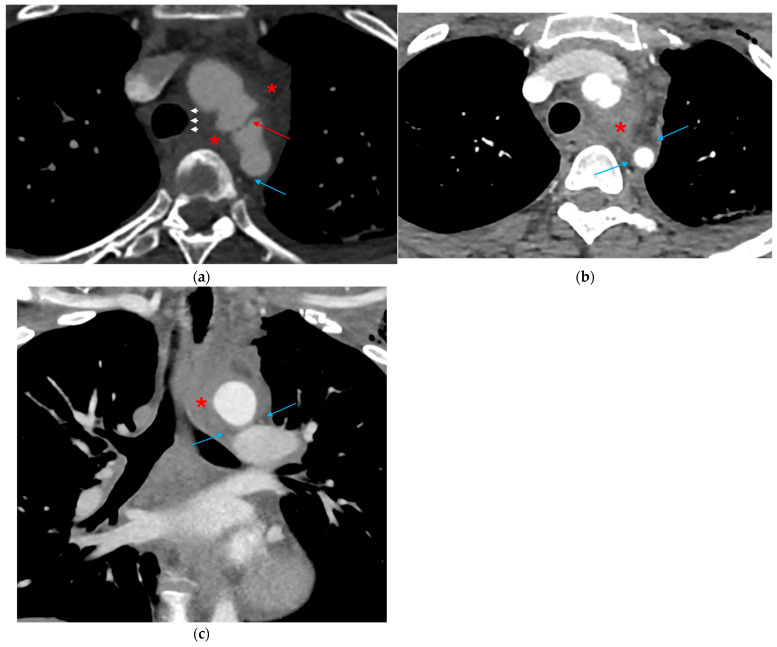
Traumatic aortic injury with periaortic hematoma. (**a**,**b**) Axial and (**c**) coronal CTA images show an intimal traumatic defect (red arrow) with associated periaortic fat stranding (blue arrows), periaortic hematoma extending across the superior, medium and posterior mediastinum (red asterisks), and right deviation of trachea (white arrowheads).

**Figure 7:**
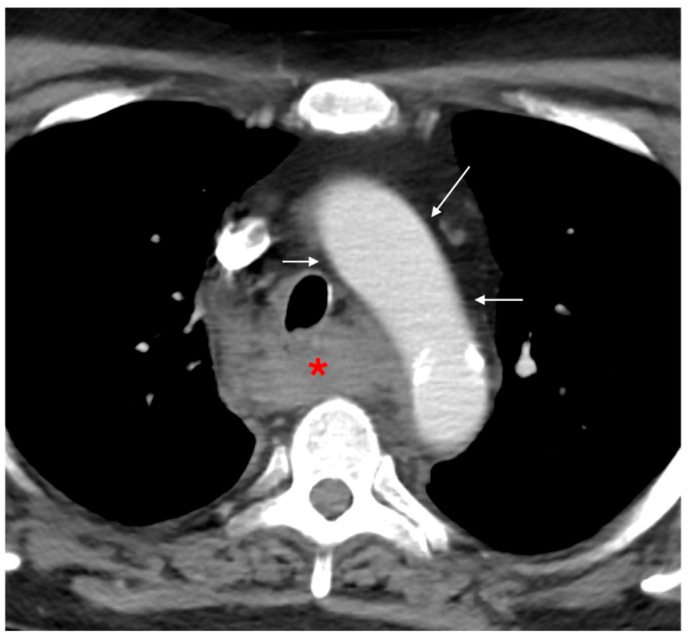
Mediastinal hematoma with preserved fat plane. Axial CTA image demonstrates a middle/visceral mediastinal paraesophageal hematoma (red asterisk) resulting from esophageal injury. The periaortic fat plane remains preserved (white arrows), without surrounding stranding, arguing against the aorta as the source of the mediastinal hematoma.

Indirect signs are findings outside the aorta; they may represent the only abnormal findings in some thoracic injuries and in most abdominal aortic injuries. A *mediastinal hematoma* appears as high-attenuation mediastinal soft tissue or blood that often tracks along mediastinal compartments. It indicates bleeding from the aorta or from other mediastinal structures; therefore, it has low specificity [10,20]. A *periaortic hematoma* is much more concerning for ATAI. This hematoma typically lies contiguous with the aortic wall, and the intervening fat plane becomes indistinct or obliterated (Figure 6). A periaortic hematoma may be related to a lesion of the vasa vasorum without frank aortic wall rupture [10]. A periaortic hematoma near the level of the diaphragm (T11-L1) has high specificity for ATAI in patients after blunt trauma [23]. At this level, however, it may also occur secondary to posterior diaphragmatic rupture or vertebral body or rib fractures [24]. If a hematoma is detected, careful assessment of the periaortic fat for stranding is warranted. The presence of an intact, clear fat plane around the aorta strongly suggests that the hematoma is unlikely to originate from an aortic injury (Figure 7) [25].

**Figure 8:**
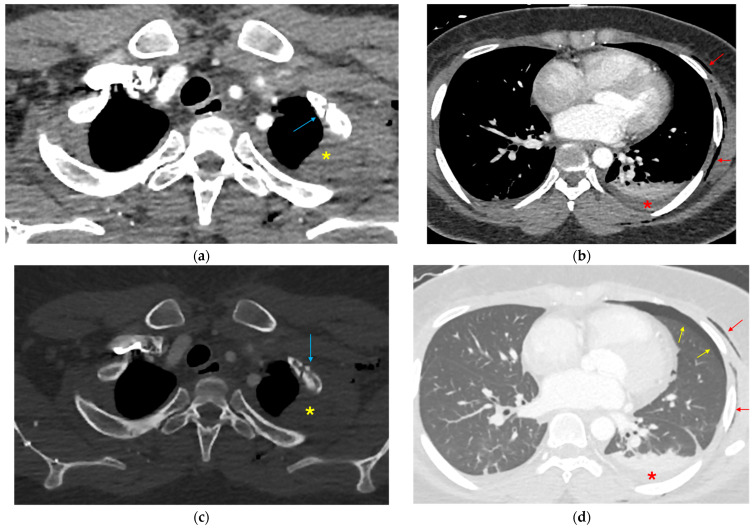
Multiple indirect signs in a patient with ATAI. (**a**,**b**) Axial soft tissue, (**c**) bone and (**d**) lung window CTA images show indirect signs of aortic injury, including left-sided first rib fracture (blue arrows), with adjacent pleural/extrapleural hematoma (yellow asterisks), left hemothorax (red asterisks), pneumothorax (yellow arrows) and subcutaneous emphysema (red arrows).

*Fractures of the sternum and the first and second ribs* are also indirect signs of severe trauma and should prompt careful evaluation of vascular structures due to their association with an increased risk of ATAI (Figure 8). Other indirect signs of ATAI include left hemothorax, pneumothorax, mediastinal deviation, obliteration of the aortic contour, thoracic or lumbar vertebral body fractures, retroperitoneal hematoma, and periaortic fat stranding [15,19,20,25]. These indirect signs should raise suspicion and prompt detailed evaluation of the aorta, particularly when multiple findings are present or when they accompany direct signs.

#### 3.1.3. Mimics and Anatomic Variations

Several normal anatomic variants and adjacent mediastinal structures may mimic ATAI on CTA, particularly at the aortic isthmus and along the origins of branch vessels. Recognition of these entities and systematic evaluation for associated direct and indirect signs of trauma are essential to avoid false positive diagnoses.

**Figure 9:**
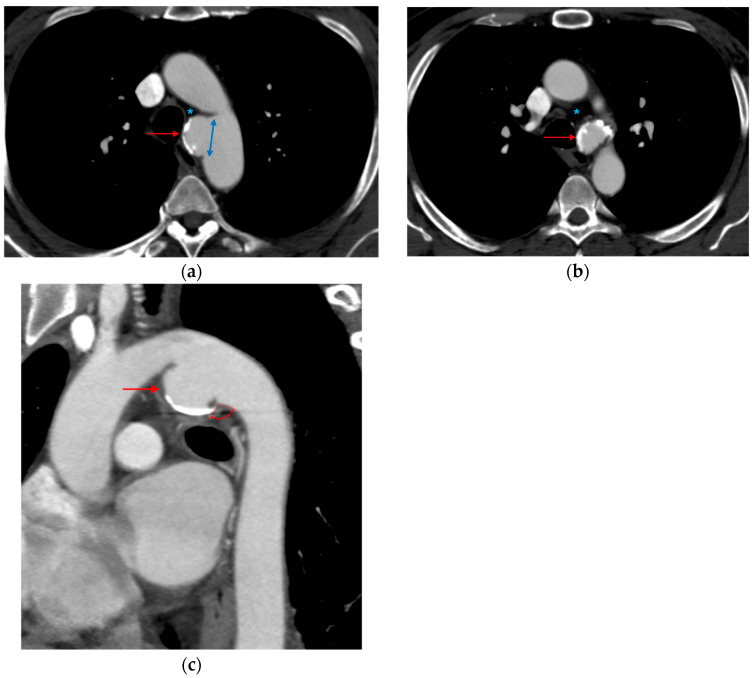
Ductus diverticulum aneurysm. (**a**,**b**) Axial CTA images show a well-defined smooth outpouching (red arrows) with a wide base (blue double arrow) and peripheral calcification with preservation of surrounding fat planes (blue asterisk). (**c**) The sagittal CTA image localizes the outpouching arising from the distal transverse aortic arch at the level of the ligamentum arteriosum (red arrow). The structure shows obtuse angles (red dotted curved line) between the outpouching and the distal aortic wall. There is no evidence of mediastinal/periaortic hematoma or an intimal disruption, which suggests a ductus diverticulum rather than a pseudoaneurysm.

*A ductus diverticulum* is a congenital developmental remnant at the site of the closed ductus arteriosus and typically appears as a smooth, broad-based focal bulge along the inferior or anteromedial aspect of the aortic isthmus, near the ligamentum arteriosum. It may appear rounded, forming obtuse angles with the aortic wall, or less commonly spiculated with more acute angles. Findings that favor a ductus diverticulum include smooth margins, a wide base, and obtuse angles between the outpouching and the adjacent aortic wall, calcification, and the absence of associated mediastinal hematoma or periaortic fat stranding (Figure 9) [15,26,27]. In the context of high-energy deceleration trauma, however, an irregular bulge at the isthmus should raise concern for a traumatic pseudoaneurysm [28].

*An aortic spindle* is a benign anatomic variant characterized by smooth, fusiform enlargement of the proximal descending thoracic aorta, usually just distal to the aortic isthmus [15,26]. Features that favor an aortic spindle include gradual symmetric dilation, smooth walls, and the absence of focal saccular outpouching or intimal disruption.

*Infundibula* and normal branch vessel ostial contours may produce small focal outpouchings that can mimic pseudoaneurysms on axial images, particularly at the origins of the supra-aortic branches of the aortic arch or at the origin of the bronchial or intercostal arteries [26]. Findings favoring a benign infundibulum include a conical or funnel-shaped morphology with a branch vessel arising from the apex, best demonstrated on multiplanar reformations (MPR) or maximum intensity projection (MIP) reconstructions, and the absence of adjacent hematoma.

**Figure 10:**
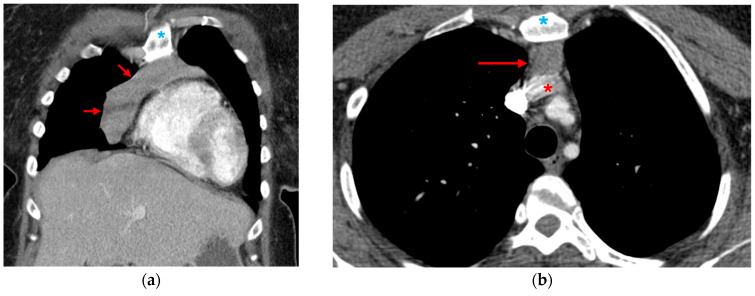
Thymic hyperplasia. (**a**,**b**) Axial CTA images show a mediastinal, homogenous soft tissue density (red arrow) positioned in the anterosuperior mediastinum immediately anterior to the aortic arch (red asterisk) behind the manubrium (blue asterisks). The location, characteristics, and smooth lobulated margins are consistent with thymic hyperplasia.

*Residual thymic tissue* or *thymic hyperplasia*, particularly in younger patients, may mimic mediastinal hematoma in the trauma setting. Preservation of the expected anterior mediastinal thymic configuration, typically with smooth margins and often relatively homogeneous attenuation, favors thymic tissue (Figure 10), whereas mediastinal hematoma is more often ill-defined and tracks along mediastinal spaces [15].

**Figure 11:**
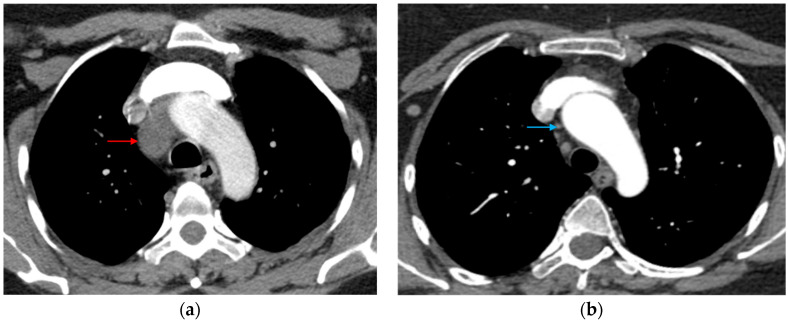
High-riding aorta superior pericardial recess. (**a**) Axial CTA image demonstrates a lobulated fluid attenuation structure in the right upper paratracheal region (red arrow) corresponding to a high-riding superior pericardial recess. (**b**) A comparative axial CTA image at the same level in a patient with no pericardial recess abnormalities (blue arrow).

*Pericardial recesses* may also simulate periaortic fluid collections or hematoma. Typically, the superior pericardial recess runs along the right side of the ascending aorta up to the sternal angle. Occasionally, part of this recess may extend upward into the upper right paratracheal area as a triangular, crescent-shaped, or elliptical water attenuation structure. This is known as a high-riding superior pericardial recess (Figure 11) [29]. These recesses typically demonstrate fluid attenuation and can be distinguished by their usual location in known pericardial spaces, their contiguity with adjacent pericardial spaces on MPR, and the maintenance of a distinct fat plane separating them from the aortic wall [16,29].

#### 3.1.4. Protocol Considerations on CT Angiography for Aortic Injury

CTA protocols for ATAI are not fully standardized and may vary depending on scanner capabilities, institutional resources, and trauma workflows. Nevertheless, protocol optimization is critical, as ATAI is a time-sensitive, potentially fatal condition that requires a balance between rapid acquisition and adequate diagnostic detail.

In the emergency trauma setting, CTA examinations should ideally be performed with intravenous iodinated contrast using a high-resolution, multiphasic protocol. CTA performed without and with contrast is endorsed by the American College of Radiology (ACR) as the most effective routine imaging modality for detecting thoracic injuries caused by blunt trauma [30]. Moreover, current European Society for Vascular Surgery (ESVS) and American College of Cardiology/American Heart Association (ACC/AHA) guidelines recommend dual-phase (arterial and venous) CTA for the initial evaluation of suspected ATAI [2,16]. This approach is particularly important because ATAI may be clinically occult on presentation and only become evident after the onset of shock or hemodynamic instability. Dual-phase imaging reduces the risk of underestimation and improves diagnostic confidence [31].

Arterial-phase images are essential for identifying direct signs of injury such as intimal flaps, pseudoaneurysm, and active contrast extravasation [32]. The venous phase aids in differentiating contained vascular injuries from actively bleeding lesions and improves the assessment of mediastinal hematoma and associated thoracic injuries [31]. Automated bolus tracking is recommended, with the region of interest typically placed in the aortic arch. The optimal threshold for triggering arterial-phase acquisition is 130–150 HU at 100–120 peak kilovoltage (kVp), depending on body habitus. Material attenuation increases at 100 kVp compared with 120 kVp. A venous phase is commonly acquired 60–70 s after contrast injection [31,33]. When feasible, contrast injection from the right upper extremity is preferred because it reduces streak artifacts from dense contrast within the left brachiocephalic vein as it crosses the aortic arch, which can degrade the assessment of the thoracic aorta [33].

Following image acquisition, advanced post-processing is strongly recommended. Thin-section reconstructions (approximately 1-mm slice thickness and reconstruction interval), along with MPR, curved planar reformats (CPR), MIP, and three-dimensional volume-rendered images, enhance lesion detection, delineate injury extent, and assist in planning endovascular repair [16,31,34]. Sagittal reformations allow rapid assessment of the entire thoracic aorta, while careful review of axial images remains essential for detecting subtle direct injuries. MIP reconstructions may increase conspicuity of subtle luminal abnormalities and facilitate assessment of longitudinal and branch vessel extension, while volume-rendered images provide an intuitive three-dimensional depiction of arch anatomy and landing-zone relationships for endovascular repair planning.

When feasible, real-time radiologist review while the patient remains on the CT table is encouraged, as it may prompt acquisition of additional phases or targeted reconstructions if equivocal findings are identified.

#### 3.1.5. CT Angiography Artifacts

**Figure 12:**
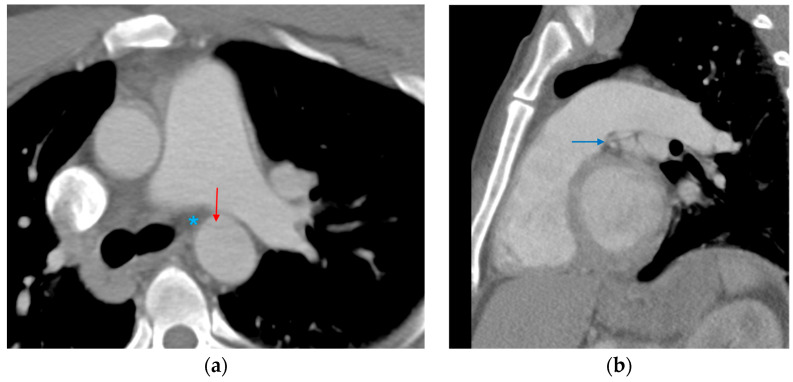
Motion artifact in the isthmus. (**a**) Axial CT image shows an artifact at the aortic isthmus simulating an intimal defect (red arrow). Please note that the periaortic fat is normal without stranding (blue asterisk). (**b**) The sagittal CTA image confirms it is a linear artifact, not a true defect (dark blue arrow).

CTA assessment of ATAI is susceptible to patient-related and technical artifacts. Motion artifacts may result from cardiac pulsation, respiratory motion, or patient movement and are common in trauma patients because of arrhythmias, pain, respiratory distress, or altered mental status. When suspected, reviewing thin-section images with MPR and short-slab MIP can help demonstrate the absence of a continuous intimal flap and avoid false positive interpretation. Sagittal reconstructions are particularly useful because they may reveal nonphysiologic artifactual step-offs (Figure 12) [11,15]. Nevertheless, repeating imaging with ECG-gated or ultrafast high-pitch acquisition (gantry speed < 0.5 s) is often the most reliable solution and should be considered when findings remain equivocal [11,33].

**Figure 13:**
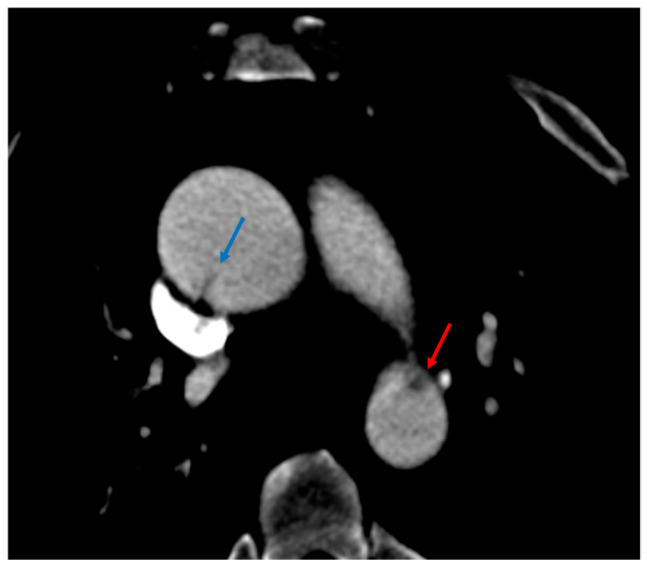
Streak artifact related to contrast beam hardening. Axial CT image shows an artifact produced by contrast within the superior vena cava at the ascending aorta simulating an intimal defect (blue arrow). A true intimal defect can be seen as a small filling defect adherent to the descending aortic wall (red arrow).

**Figure 14:**
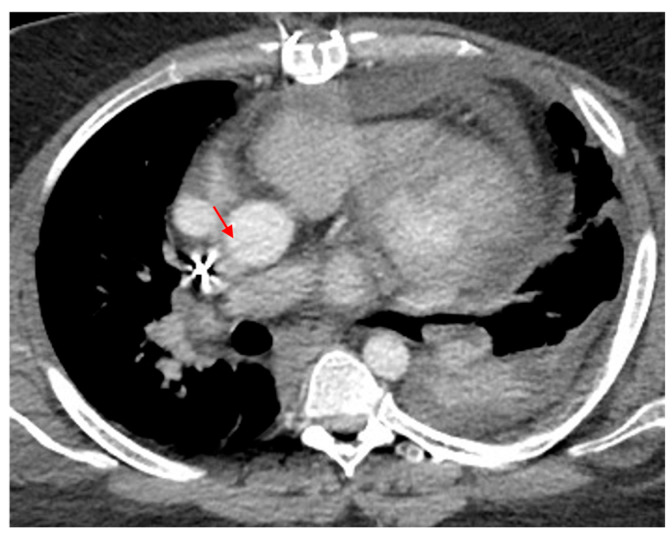
Metal artifact. Axial cardiac CT image shows a metallic artifact (red arrow) from a central venous catheter.

**Figure 15:**
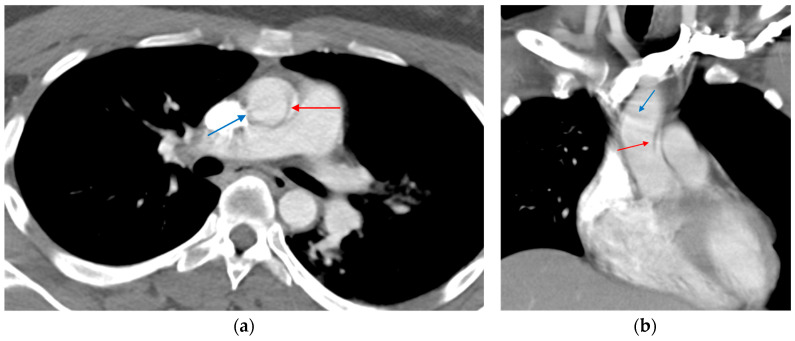
Pulsation artifact and streak artifact related to contrast beam hardening. (**a**) Axial and (**b**) coronal CTA images of the chest demonstrate a curvilinear intraluminal filling defect in the ascending aorta (red arrow), compatible with pulsation artifact and mimicking an intimal flap. Dense contrast within the superior vena cava produces an adjacent streak artifact (blue arrow) that further degrades the evaluation of the ascending aorta.

Other technical artifacts on CTA arise from contrast and include beam-hardening streak artifacts, suboptimal contrast timing, and flow-related artifacts from inadequate bolus delivery or arterial mixing (Figure 13). Metal artifacts result in a characteristic streak artifact composed of intervening bright and dark streaks (Figure 14). Different artifacts may coexist, further degrading the evaluation of the aorta (Figure 15). Recommendations for recognizing and avoiding or reducing these artifacts are shown in Table 1.

#### 3.1.6. ECG-Gated CT Angiography

As discussed in the artifacts section, various factors may compromise image quality, potentially resulting in misdiagnosis. For hemodynamically stable trauma patients, ECG-gated CTA is highly recommended to minimize motion artifacts and enhance the visualization of intimal injuries [2].

The principal advantage of ECG-gated imaging is improved detection of intimal flaps and more precise anatomic delineation for endovascular planning, including stent-graft sizing and access strategy. ECG-gated CTA demonstrates higher diagnostic accuracy, improved interobserver agreement, and greater reader confidence compared with non-gated acquisitions, regardless of reader experience in other acute aortic diseases [40]. In contrast, non-gated studies often yield suboptimal visualization, even with MPR [18].

ECG-gated CTA may be performed using prospective or retrospective acquisition. Prospective gating captures images at a single cardiac phase, whereas retrospective gating acquires data throughout the cardiac cycle and has been associated with higher radiation exposure. However, a 70% dose reduction has been reported at 80 kVp, yielding exposure levels comparable to those of prospective acquisition [41]. Despite the overall increase in radiation exposure, the improved detection of intimal tears with full-phase retrospective images may justify its use in acute settings, where missed diagnoses can be fatal [42].

Another important consideration for ECG-gated CTA is heart rate. Current recommendations suggest that a heart rate above 60 beats per minute is required to perform the study, a threshold that may be difficult to achieve in the trauma setting [43]. However, this recommendation has been revisited in light of advances in CT technology. Evidence indicates that prospectively gated CTA can still be performed at heart rates exceeding 65 beats per minute on 128-slice or higher scanners, where temporal resolution is sufficient [44].

Despite guideline endorsement, ECG-gated CTA is inconsistently implemented in trauma workflows due to practical constraints, including longer acquisition times, additional radiation exposure, breath-hold requirements, limited scanner availability, and the need for technologist expertise [45,46].

In addition, evidence demonstrating that ECG-gated CTA consistently alters clinical management or outcomes in patients with borderline or indeterminate ATAI remains limited, contributing to substantial variability in institutional practice and a persistent gap between technical capability and real-world implementation. When ECG gating is not feasible, high-pitch or ultrafast acquisition protocols provide a viable alternative to minimize motion artifacts while preserving diagnostic accuracy [32].

#### 3.1.7. Emerging CT Technologies

Advanced CT technologies are increasingly being explored in vascular imaging; however, their role in the acute evaluation of traumatic aortic injury remains limited, and current evidence specific to ATAI is sparse.

*Cinematic rendering* is an advanced three-dimensional visualization technique that produces photorealistic representations of the vascular and surrounding structures. While it does not provide additional diagnostic information beyond conventional reconstructions, it may enhance anatomic understanding in complex or equivocal cases and facilitate multidisciplinary communication in selected scenarios [47]. Its role in ATAI is therefore considered adjunctive rather than diagnostic.

*Spectral CT techniques*, including dual-energy CT (DECT) and photon-counting CT (PCCT), offer theoretical advantages in vascular imaging [45]. Nevertheless, they are not currently included in routine ATAI assessments and are not yet available in every center.

*DECT* enables acquisition at multiple energy levels, allowing for differentiation between iodine, thrombus, and calcification, as well as the generation of virtual non-contrast images and low-keV reconstructions. These features may improve contrast conspicuity and reduce metal and beam-hardening artifacts, particularly following endovascular repair [48]. Although DECT has not been explicitly validated for ATAI, studies in other acute bleeding scenarios and in post-endovascular surveillance have demonstrated improved detection of active hemorrhage and endoleaks, suggesting potential applicability in selected trauma settings [49,50].

*PCCT* is an emerging detector-based technology that directly counts individual photons and measures their energy, thereby improving spatial resolution, reducing image noise, and providing intrinsic spectral information. Early studies in post-endovascular aortic repair have shown improved visualization of stent grafts and vessel lumen compared with conventional CT [51,52]. However, its availability remains limited, and no studies to date have evaluated its diagnostic or clinical impact in acute traumatic aortic injury.

### 3.2. Chest MR Angiography

Magnetic resonance angiography (MRA) is not routinely used for the initial diagnosis of ATAI and has a limited role in the trauma setting due to longer acquisition times, patient instability, and restricted availability. However, it serves as a valuable alternative or adjunctive modality during follow-up and long-term surveillance in selected patients with MRI-compatible stent grafts, as endorsed by current ESVS guidelines [16]. MRA allows for the assessment of aortic morphology, intramural hemorrhagic components, and post-intervention complications without exposure to ionizing radiation or iodinated contrast, particularly in younger trauma patients who may be at increased long-term risk from cumulative ionizing radiation exposure [11,16].

Advanced techniques, including 4D flow MRI, have been used to evaluate complex aortic hemodynamics following endovascular repair, providing insight into flow patterns, wall shear stress, and aortic stiffness [53,54,55]. These parameters may be particularly relevant in younger patients, in whom increased aortic stiffness has been associated with ventricular hypertrophy, arterial hypertension, and adverse long-term cardiovascular outcomes following ATAI.

Despite these advantages, MRA remains complementary rather than a replacement for CTA, and its use is generally limited to stable patients with MRI-compatible endografts or specific clinical indications.

## 4. Classifications

**Figure 16:**
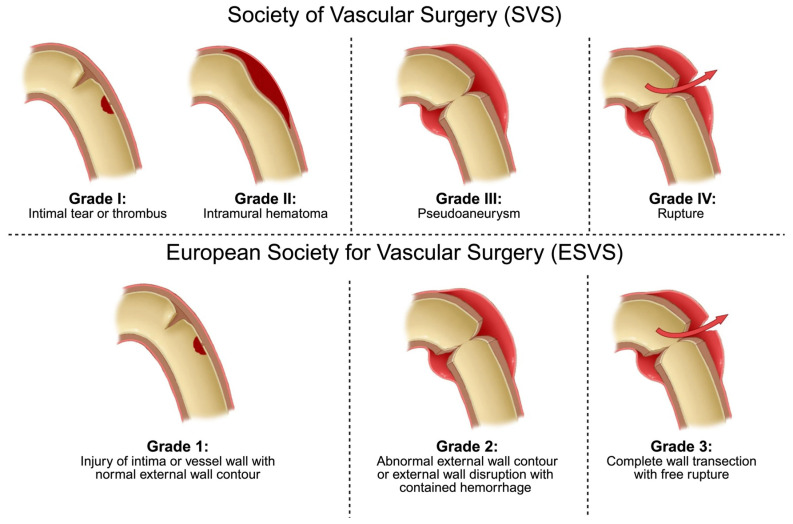
Comparison of the Society for Vascular Surgery (SVS) and European Society for Vascular Surgery (ESVS) classification systems for traumatic aortic injury. The illustration demonstrates the correspondence between the two systems: SVS grades I and II correspond to ESVS grade 1, SVS grade III corresponds to ESVS grade 2, and SVS grade IV corresponds to ESVS grade 3. Red arrows indicate the site and direction of wall disruption/free rupture in the highest-grade injuries. Source: This figure was created by the authors.

Traumatic aortic injuries range from minor contusions to complete transection, which is known as aortic rupture. The most widely used system for classifying ATAI is the one adopted by the Society for Vascular Surgery (SVS). This system classifies injuries according to the extent of damage to the layers of the aortic wall [2,5]. Subsequently, multiple classification systems were developed to incorporate injury morphology and extent [56,57,58,59]; however, these have not been adopted as widely as the SVS classification. More recently, the ESVS guidelines (2025) introduced refinements that incorporate lesion morphology and imaging risk markers to guide management [16,60]. These frameworks aim to stratify patients more effectively, with the potential to reduce overtreatment. The ESVS classification is broadly consistent with the SVS classification (Figure 16).

## 5. Treatment Modalities

**Figure 17:**
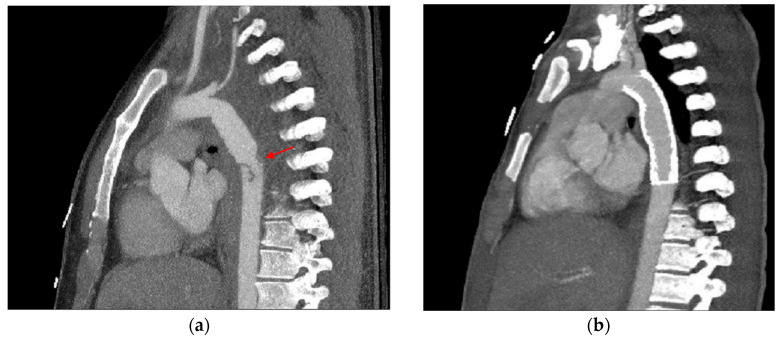
Thoracic endovascular aortic repair. (**a**) Sagittal CT image of a patient with grade III ATAI (red arrow). (**b**) Sagittal CT image following successful TEVAR.

Most international societies advocate endovascular repair as the first-line treatment for ATAI in patients with suitable anatomy [2,5]. Thoracic endovascular aortic repair (TEVAR) involves placing a stent graft within the aorta to isolate the damaged area and restore normal blood flow (Figure 17). Reported success rates of TEVAR range from 98–100%, with a re-intervention rate of 1.0% [61,62,63] and lower rates of perioperative complications and mortality than open repair [61,62]. In cases in which aortic anatomy is not compatible with stent graft placement, open thoracic repair is performed, whereby the damaged aorta is replaced with a synthetic graft [16].

## 6. The Role of Imaging in Management

The management of ATAI, including endovascular stent-graft placement or nonoperative measures, depends on the severity and location of the injury. SVS grade I and II lesions (ESVS: grade 1) are typically managed conservatively with regular CTA follow-up until resolution of the aortic lesion. Real-world practice patterns in the management of SVS grade II injuries have been heterogeneous. Current data support nonoperative management and imaging surveillance for grade II blunt traumatic aortic injury instead of endovascular repair [64]. SVS grade III (ESVS: grade 2) lesions are treated based on imaging findings and high-risk features. Low-risk ATAI features allow delayed (>24 h) repair and stabilization of other injuries before aortic repair. Lesions with high-risk features require urgent (<24 h) endovascular or open repair. SVS grade IV (ESVS: grade 3) lesions require emergency repair because of their higher risk of complications [2,5].

### 6.1. Imaging Characteristics Relevant to Management

#### 6.1.1. High-Risk Imaging Features

The assessment of injury severity and the identification of high-risk imaging features are critical for triage and management decisions. While lower- and higher-grade injuries follow relatively established management pathways, high-risk features influence treatment decisions for intermediate-grade injuries. High-risk imaging features endorsed by the ACC/AHA and ESVS guidelines are summarized in Table 2.

Although not routinely measured, aortic arch morphologic parameters may provide additional risk stratification and predict technical challenges during endovascular repair [8,65]. *The aortic arch index* is measured as the distance from the outer wall of the ascending aorta to the outer wall of the descending aorta on the lesser curvature in the horizontal plane, at the height of the mid-left bronchus. *The aortic arch angle* is obtained by measuring the angle formed by connecting three points: (1) the outer wall of the ascending aorta (level of mid-left bronchus); (2) the highest point on the outer wall of the aortic arch; and (3) the outer wall of the descending aorta at the level of the mid-left bronchus. Both a lower aortic arch index and a more acute aortic angle are associated with greater ATAI severity [65].

#### 6.1.2. Role of Imaging in Preoperative Endovascular Planning

**Figure 18:**
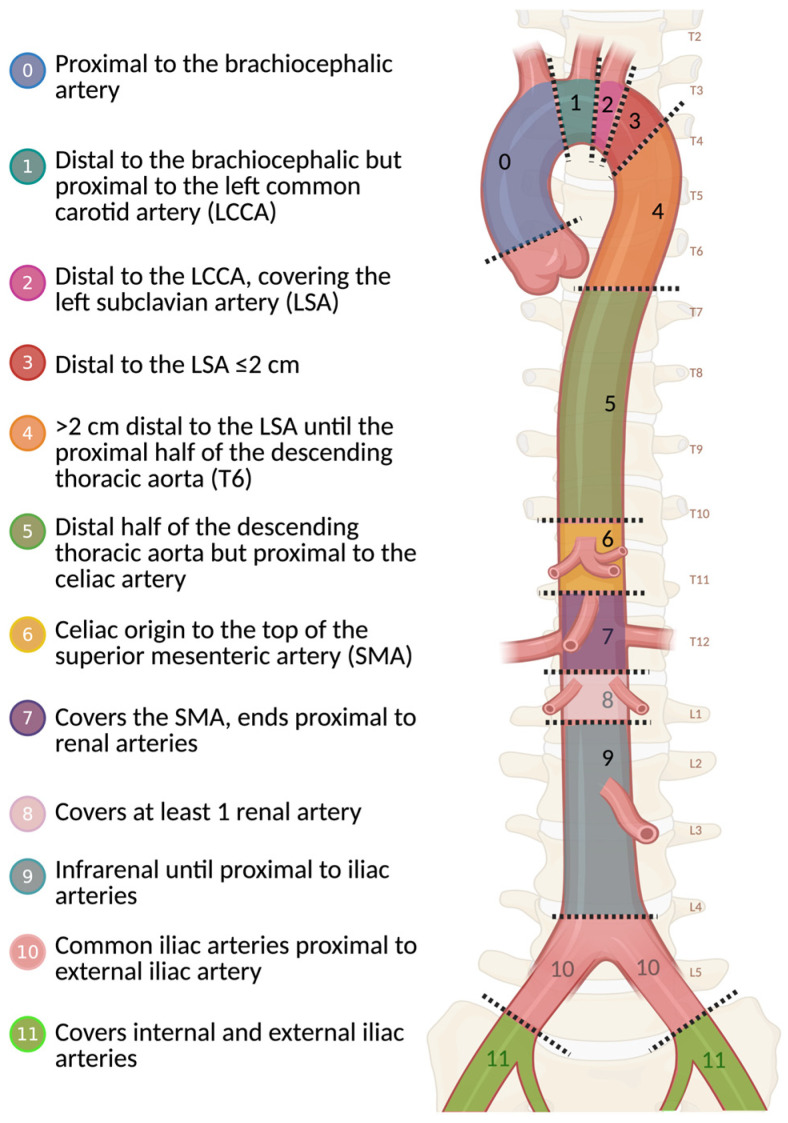
Aortic landing zone. Illustration of Ishimaru aortic zones, later adopted and standardized by the Society for Vascular Surgery (SVS) for endovascular repair. Source: This figure was created by the authors.

Accurate reporting of injury location, length, and preoperative CTA measurements, including proximal and distal aortic diameters and the lesion’s relationship to branch vessels, is essential for endovascular planning. Injury location is best described using the Ishimaru aortic zones, later adopted and standardized by the SVS for TEVAR (Figure 18) [66,67]. Attachment zone location influences procedural complexity, device selection, and mechanical stress on the stent graft [67]. Injuries involving zones 0–2 (the ascending aorta and transverse arch) are rare among patients who reach the hospital alive due to rapid fatal hemorrhage [16]. Zone 9 (the abdominal aorta) has been identified in autopsy studies as a region with a high rate of potentially preventable deaths, warranting careful evaluation to avoid missed diagnoses [16]. Reporting the orthogonal diameters of the normal aorta at the proximal and distal landing zones is necessary, as these measurements guide endograft sizing and oversizing [68]. In cases of hemodynamic instability, a marked reduction in aortic diameter has been observed despite adequate resuscitation [69]. In young trauma patients, reporting the aortic root diameter may help provide a more reliable reference for endograft sizing [68]. Other measurements, such as *the isthmus angle* (defined as the angle between the horizontal aortic arch and the descending aorta), have been reported to predict significant narrowing during the follow-up period after TEVAR [70].

## 7. Post-Treatment Imaging Surveillance and Complications

Imaging plays a central role in the post-treatment management of ATAI, as both endovascular and open surgical repair are associated with complications that are primarily detected during routine surveillance. Radiologists are therefore integral to identifying early technical failures and delayed graft- or surgery-related sequelae that may directly impact patient outcomes.

### 7.1. Imaging Modality and Surveillance Strategy

CTA remains the primary imaging modality for post-treatment surveillance after both endovascular and open repair of ATAI, given its wide availability, rapid acquisition, and high spatial resolution. MRA is a valuable alternative. However, the optimal imaging surveillance strategy following ATAI repair has not been definitively established and should be individualized based on lesion characteristics, treatment modality, and patient-specific factors.

For patients with low-grade ATAI managed non-operatively, most lesions demonstrate complete resolution, improvement, or stability on follow-up imaging [11]. Current recommendations suggest repeat CTA within 48–72 h to assess lesion stability [16]. Imaging findings such as a new or enlarging aortic outpouching, progression of an intimal-medial flap, new contrast extravasation, vessel occlusion, or changes in aortic caliber indicate instability and warrant consideration for repair [2,71].

Long-term surveillance protocols should be determined on a case-by-case basis, with recommended durations summarized in Table 3 [2,16,71]. Surveillance after TEVAR may reduce mortality by identifying short term complications, such as unsuccessful exclusion and endograft infolding and long term complications, such as graft-related problems such as endoleak, collapse, and migration. Although surveillance is essential for detecting early unsuccessful exclusion and graft-related complications, the optimal long-term imaging strategy after TEVAR remains uncertain. Available long-term data suggest that most procedure-related complications occur within the first year, raising ongoing questions regarding the duration, interval, and modality of lifelong surveillance, particularly in younger patients who undergo repeated CTA.

### 7.2. Complications Following Endovascular Repair

Following TEVAR, several complications may arise that require systematic imaging follow-up. Among the most relevant are stent-induced new entry (SINE) and distal stent-induced new entry (dSINE), which result from new intimal disruptions at the proximal or distal margins of the stent graft. On follow-up CTA or completion angiography, these entities may appear as focal intimal defects, contrast outpouchings, or persistent false lumen perfusion adjacent to the stent edges. SINE typically develops 12–36 months after TEVAR and is frequently asymptomatic; it is most often detected on routine surveillance imaging, highlighting the importance of follow-up in this population [72].

Additional imaging-detectable complications after TEVAR include endograft collapse, narrowing, infolding, migration, and endoleak, all of which may compromise effective aortic exclusion and necessitate prompt recognition during both early and late surveillance [71]. Endograft collapse represents one of the most severe complications and has been reported to occur at a median of 15 days following TEVAR, underscoring the need for early post-procedural imaging [73].

### 7.3. Complications Following Open Surgical Repair

Open surgical repair is associated with a distinct complication profile. While many adverse events are primarily clinical, imaging remains essential for detecting sequelae such as spinal cord ischemia, renal ischemic injury, and postoperative vascular or paraspinal complications, particularly when new neurologic deficits or renal dysfunction develop [61,62]. Early identification of these complications on imaging is critical, as timely diagnosis may alter management and improve outcomes.

## 8. Conclusions

ATAI is a life-threatening condition that requires prompt and accurate diagnosis. CTA remains the diagnostic cornerstone, enabling the detection of direct signs of injury, including an intimal flap, intramural hematoma, pseudoaneurysm, and contrast extravasation. Indirect findings such as periaortic hematoma, left first rib fracture, hemothorax, and periaortic fat obliteration or stranding should heighten suspicion for ATAI and prompt careful evaluation of the aorta. In real-world trauma practice, however, interpretation may be complicated by mimics, anatomic variants, suboptimal CTA technique, and patient-related or technical artifacts. CTA should ideally be performed with intravenous iodinated contrast using a high-resolution, multiphasic protocol with multiplanar and three-dimensional reconstructions. Rapid communication between radiologists and treating teams, along with familiarity with ATAI imaging findings and severity classifications, is essential to ensure accurate and timely diagnosis and guide effective emergency management. After treatment, imaging surveillance remains critical for detecting early technical failure and delayed graft- or surgery-related sequelae that may directly affect patient outcomes.

## Figures and Tables

**Table 1:** Technical Artifacts for Aortic Injury imaging.

Artifact	How to Recognize	How to Avoid
Pulsation/cardiac motion	Curvilinear pseudoflap, stair-step artifact, blurring, ghosting, dark bands; greatest in the ascending aorta	ECG-gated CTA; high-pitch acquisition; shorter acquisition time; motion correction algorithms
Breath-hold/respiratory motion	Misregistration, double contours, blurring of the aortic wall or pulmonary vasculature, rib or sternal motion	Proper breath holding when feasible; shorter acquisition time; repeat acquisition if needed
Contrast beam hardening	Bright dark streaks extending from dense contrast in the SVC, brachiocephalic vein, or right atrium.	Saline flush; biphasic/triphasic injection; right-arm injection when feasible; higher-energy acquisition; reconstruction correction
Suboptimal contrast timing	Inadequate or heterogeneous aortic opacification, layering, poor target vessel enhancement, failed triggering	Correct ROI placement; test bolus when needed; adequate intravenous access and injection rate
Metal	Bright and dark streaks adjacent to metallic material	If single-energy scan: Higher tube potential; If dual-energy scan: virtual high-monoenergetic reconstructions
Image noise (quantum mottle)	Irregular grainy appearance obscuring subtle aortic wall findings.	Increase tube current or voltage; thicker reconstructions; iterative reconstruction; patient repositioning

ROI: region of interest; SVC: superior vena cava. Source [35,36,37,38,39].

**Table 2:** High-risk imaging features for acute traumatic aortic injury.

ACC/AHA Feature [2]	ESVS Feature [16]	Comment
Posterior mediastinal hematoma > 10 mm	Large mediastinal hematoma	Same concept; ESVS does not specify posterior or size.
Mediastinal hematoma causing mass effect		Overlaps with slightly broader ESVS description.
Large left hemothorax	Left hemothorax	Both highlight left-sided hemothorax as high risk.
Pseudocoarctation of the aorta	Aortic coarctation	Same pathophysiologic risk; slightly different terminology.
Lesion-to-normal aortic diameter ratio > 1.4	Large pseudoaneurysm	Lesion can represent a pseudoaneurysm; imaging-based criterion.
Ascending aortic, aortic arch, or great vessel involvement	—	Unique to AHA; highlights proximal aortic involvement.
Aortic arch hematoma	—	Unique to AHA; specific to arch involvement.

ESVS: European Society for Vascular Surgery; ACC/AHA: American College of Cardiology/American Heart Association. Source [2,16].

**Table 3:** Follow-up recommendations in accordance with international guidelines.

Treatment Type	ESVS 2025 [16]	ACC/AHA 2022 [2]	SVS 2018 [71]
Open surgical repair	- CTA or MRA at 1 year - MRA at 5 years	- CT or MRI at 1, 6, and 12 months - Then annually if stable	- CT (±contrast) every 5 years
Endovascular repair (TEVAR)	- CTA or MRA at 1 month - MRA at 1 year - MRA continued for at least 5 years	- CT or MRI at 1, 6, and 12 months - Then annually if stable	- Contrast-enhanced CT at 1 and 12 months, then annually - If abnormal at 1 month → repeat at 6 months - Longer intervals once stability is confirmed
No repair	- MRA at 1 month - Yearly MRA until complete remodeling	- CT at 1, 6, and 12 months - If stable: at appropriate intervals (depending on injury type and extent)	No specific recommendation

ESVS: European Society for Vascular Surgery; AHA: American Heart Association, TEVAR: thoracic endovascular aortic repair. Source [2,16,71].

## Data Availability

No new data were created or analyzed in this study.

## Abbreviations

The following abbreviations are used in this manuscript:

ACC/AHA	American College of Cardiology/American Heart Association
ATAI	Acute Traumatic Aortic Injury
CT	Computed Tomography
CTA	Computed Tomography Angiography
DECT	Dual-energy CT
ECG	Electrocardiogram
ESVS	European Society for Vascular Surgery
IMH	Intramural Hematoma
MRA	Magnetic Resonance Angiography
MRI	Magnetic Resonance Imaging
PCCT	Photon-Counting CT
SVS	Society for Vascular Surgery
TEVAR	Thoracic Endovascular Aortic Repair
